# Genomic evolution and climate related drivers of cholera surges in Dhaka Bangladesh between 1996 and 2024

**DOI:** 10.1038/s43856-026-01620-8

**Published:** 2026-05-22

**Authors:** Md Mamun Monir, Khalilur Rahman, Mohammad Tarequl Islam, Marzia Sultana, Wali Ullah, Sardar Muhammad Osail, Md Alimul Islam Ridoy, Abdullah Al-Mamun, Shirajum Monira, Kimberley D. Seed, Andrew Camilli, Carla Mavian, Nicholas Thomson, Rita R. Colwell, ASG Faruque, Tahmeed Ahmed, Munirul Alam

**Affiliations:** 1 icddr,b, International Centre for Diarrhoeal Disease Research, Bangladesh, Dhaka, Bangladesh; 2 Department of Plant and Microbial Biology, University of California, Berkeley, Berkeley, CA USA; 3 Department Molecular Biology and Microbiology, Tufts University, Boston, MA USA; 4 Emerging Pathogens Institute, Department of Pathology, College of Medicine, University of Florida, Gainesville, FL USA; 5 Parasites and Microbes Program, Wellcome Sanger Institute, Wellcome Genome Campus, Hinxton, Cambridge UK; 6 London School of Hygiene and Tropical Medicine, London, UK; 7 Cell Biology and Molecular Genetics, University of Maryland, College Park, MD USA; 8 University of Maryland Institute for Advanced Computer Studies, College Park, MD USA

**Keywords:** Computational biology and bioinformatics, Infectious-disease epidemiology, Bacterial infection

## Abstract

**Background:**

Cholera, a climate-associated diarrhea occurs endemically in Dhaka city, Bangladesh, often turning into large epidemics. In 2022, a massive outbreak struck Dhaka city, resulting in a very high number of cholera cases. Although extensive studies have focused on climate and cholera, including genomic evolution of the causative agent, *Vibrio cholerae*, their association with the case surges remains poorly understood.

**Methods:**

We analyzed cholera data between 1996 and 2024 from the Diarrheal Disease Surveillance System (DDSS) of icddr,b, and also conducted a follow up genomic analysis of *V. cholerae* O1 El Tor by including newly sequenced and archived sequence data. Integrated analyses of genomic, climatic, and cholera surveillance data investigated the role of different factors on yearly case surges in Dhaka city.

**Results:**

Analysis of DDSS data shows temporal changes of cholera cases across Dhaka city, and genomic analysis shows continuous evolution of *V. cholerae*. Association analysis of cholera case data against the genomic data reveals that co-circulation of multiple lineages/sub lineages of *V. cholerae*, as well as their flux between the years with newly introduced or re-emerged lineages, are significantly (p-value <= 0.05) associated with the yearly cholera cases in Dhaka city. Integrated statistical association analysis of cholera cases with the climate and genomic evolutionary factors also reveals a significant association.

**Conclusions:**

This study associates climate factors and the *V. cholerae* genome evolutionary events with interannual variation, such as high and low cholera, including surges of the disease in Dhaka city.

## Plain language summary

Cholera is an infection caused by consuming food or water containing *Vibrio cholerae* bacterium. It results in severe diarrhoea. There are annual seasonal outbreaks of cholera in Dhaka city, Bangladesh. However, the size of the outbreaks varies across different years. Here, we explore the relationship between different strains of *V. cholerae*, the climate and the number of cholera cases in Dhaka city. Changes in the strain and climate can result in increased numbers of cases. This study demonstrates that including information about the *V. cholerae* strain causing infections can improve the modelling of possible risk of an epidemic. This information can be used to design strategies to prevent cholera outbreaks in Dhaka city, and in similar settings around the world.

## Abstract (web summary)

Monir et al., analyze surveillance data, genomic data from *Vibrio cholerae* and regional climate data collected from 1996 to 2024 in Dhaka, Bangaledesh. Climate factors and *V. cholerae* genome evolutionary events associate with variation in incidence of cholera, including major epidemics of the disease.

## Introduction

Cholera is an ancient infectious diarrheal disease that transmits rapidly and progresses to hypovolemic shock and death if left untreated^1^. According to a report published in 2015, cholera is responsible for approximately 1.3–4.0 million cases world-wide with 21,000 to 143,000 deaths each year^2^. Cholera remains one of the major public health challenges in low and middle-income countries (LMICs) of Asia and Africa, where the disease has long been endemic and often turns into large epidemics killing and affecting millions^2–4^. Bangladesh is the ancient home and one of the major global hotspots of cholera. Dhaka, the capital city of Bangladesh, experiences recurrent seasonal outbreaks of cholera with a characteristic dual peak pattern for over many decades^5^. According to the WHO Global Task Force on Cholera Control (GTFCC), an estimated 109,052 cholera cases occur annually in Bangladesh and with a population of ca. 66,495,209 living at an elevated risk of the disease^6^.

The causative agent of cholera, *Vibrio cholerae*, is well-adapted to the aquatic environment, and the disease transmits via drinking contaminated water. *V. cholerae* is a versatile bacterium with well over 200 serogroups, of which the serogroup O1 is responsible for endemic and epidemic cholera. Although the serogroup O139 can also cause cholera-like diarrhea, this serogroup is rarely, if ever, found associated with cholera; while the non-O1/O139 strains, collectively known as NOVC, are associated with mild-moderate diarrhea cases occurring sporadically^7^. Serogroup O1 has two biotypes, classical and El Tor of which the latter is causing the currently ongoing 7^th^ cholera pandemic by replacing the classical biotype responsible for the earlier pandemics^8^. In recent years, extensive genomic studies have been done on *V. cholerae* to characterize the bacterium^9–12^. Our comparative genomic studies showed two major lineages of *V. cholerae* O1 El Tor, BD-1 and BD-2, as they were predominantly associated with endemic and epidemic cholera in Bangladesh over the years^3^. Based on their genome phylogeny BD-2 forms part of a clade that includes strains isolated only from the Asian regions, whereas the BD-1 is part of a characteristically different lineage found distributed globally^13^. Furthermore, a subclade of the BD-1, namely BD-1.2, emerged recently, and was found associated with the massive cholera epidemic that struck Dhaka city in 2022, affecting large areas, and the patients seeking care at the icddr,b surpassed all past records of the daily patients admitted at the hospital^13^.

Studies over the decades have established a close link of cholera transmission with the climate factors such as temperature, rainfall, and humidity, showing that they can influence the incidence of cholera, supporting the survival and proliferation of *V. cholerae* bacterium in the environment^14–17^. Dhaka, with its monsoon climate and seasonal variation in the incidence of cholera, provides a unique setting to explore the interrelationships of climate factors and the disease dynamics. This study was thus undertaken to examine the dynamic changes in the number of cholera cases, their association with the transmission and evolutionary dynamics of the causative agent, *V. cholerae* O1 El Tor, and various climate factors to better understand the drivers for the cholera case surges in Dhaka city; and the aim is to aid disease control and prevention strategies.

Association analysis reveals significant association of genomic evolutionary events of *V. cholerae* O1 El Tor strains and climate factors with yearly cholera cases. Therefore, both climate and genomic evolutionary events play key roles in shaping the dynamics of disease transmission and case surges in Dhaka city.

## Materials and methods

### Cholera case and climatic data

In this study, we used data from the Diarrheal Disease Surveillance System (DDSS) of the International Centre for Diarrhoeal Disease Research, Bangladesh (icddr,b). DDSS systematically enrolled 2% of all patients, regardless of age, sex, or diarrhea severity, and recorded an extensive data bank that includes sociodemographic details, environmental history, clinical features, nutritional status, and identified diarrhea pathogens. A total of 76,815 patient data sets collected from 1996 to 2024 included 12,255 (15.95%) that proved positive for cholera. Association between number of cholera cases and recorded variables was analyzed to determine the factors contributing to increase in cases during major outbreaks. Daily temperature, relative humidity, and rainfall data in Dhaka collected between 1996 and 2024 were provided by the Bangladesh Meteorological Department.

### *Vibrio cholerae* isolates and whole genome sequencing

A total of 451 strains isolated from Bangladesh between 1991 and 2024 were analyzed to construct a phylogenetic tree and define lineages or subgroups. Among the strains, 437 were from stool samples collected under protocols reviewed and approved by the icddr,b institutional review board (Research review and ethical review committees). A total of 11 strains isolated in 2024 were newly sequenced in this study. Then, the genome sequence data of these strains were combined with our previously sequenced strains, and the collective sequenced data available for strains isolated in Bangladesh were analyzed for this study. The protocol used for sequencing was as follows: Qiagen DNA Extraction Kit was used for genomic DNA was extraction from pure cultures of *V. cholerae* isolates. NanoDrop 1000 spectrophotometer (Thermo Fisher Scientific, United States) and Qubit 4.0 fluorometer (Life Technologies) were employed for DNA quality control and quantification, respectively. Illumina DNA Prep Library Preparation Kit (Illumina) was used for preparing whole genome sequencing libraries from 300 to 350 ng of genomic DNA following manufacturer’s instructions. Sequencing was carried out at the icddr,b Genomics Center on an Illumina Miseq platform, and 150 bp paired-end sequencing reads were generated.

### Bioinformatic analysis

Sequence processing and Quality of the raw shotgun paired-end sequences were assessed using an ultra-fast quality control analysis program, fastp v0.23.2^18^. De-novo genome assembly was performed using Spades v3.15.4^19^, and assembled genomes were annotated using Prokka v1.14.5^20^. Genomic variants (SNPs, and INDELs) were called by aligning genomic reads of the sequenced strains with N16961 strain reference genome sequence (NCBI accession no. NC_002505.1 and NC_002506.1) using snippy v4.6.0^21^. Maximum likelihood phylogenetic tree was constructed using IQ-TREE v2.2.0^22^ with 1000 bootstrap and best fitted evolutionary model selected using ModelFinder^23^. Maximum clade credibility tree was conducted using BEAST v.2.6.7^24^. Maximum likelihood phylogenetic tree was visualized using iTOL^25^, and maximum clade credibility tree was visualized using ggtree R package^26^. Standalone Blast^27^ was used searching genomic islands/genes (e.g., VSP-2, ctxB alleles) within the assembled contig sequences. Impact of missense mutations was analyzed using different bioinformatic tools. The BLOSUM62 scoring matrix^28^ was used to assess the evolutionary likelihood of mutation. Grantham’s Distance^29^ measured physiochemical differences between the reference and mutant amino acid with higher scores indicating more radical (non-conservative) changes. SIFT (Sorting Intolerant From Tolerant) assessed the effect of mutation on protein function using the SWISS-PROT/TrEMBL database^30^ and DDMut^31^ to predict changes in protein stability due to missense mutation.^32^

### Statistical modelling for cholera case data

#### Weekly case data

Daily cholera case data from the 2% hospital-based surveillance and daily climatic data were converted to weekly for association analysis to ignore excessive zero counts. Temperature and relative humidity for each week were averaged, and weekly cholera cases and rainfall aggregated. A generalized additive model (GAM) was used for association analysis by considering the weekly cholera cases following a negative binomial distribution. The model is as follows:

$${{Case}}_{t} \sim {{{\rm{NB}}}}\left({\mu }_{t},\theta \right)$$

$$\log \left({\mu }_{t}\right)= \,{\beta }_{0}+s\left({{{{\rm{T}}}}}_{t}\right)+s\left({{{{\rm{R}}}}}_{t}\right)+s\left({{{{\rm{RH}}}}}_{t}\right)\\ +s\left({{{{\rm{T}}}}}_{t-1}\right)+s\left({{{{\rm{R}}}}}_{t-1}\right)+s\left({{{{\rm{RH}}}}}_{t-1}\right)$$

Where, ${{Case}}_{t}$ = cholera cases at week *t*; ${\mu }_{t}$ = expected cholera cases at week *t*; *θ* = dispersion parameter for the Negative Binomial distribution; ${\beta }_{0}$ is intercept; *s*() is function for capturing nonlinear relationships; ${{{{\rm{T}}}}}_{t}$, ${{{{\rm{R}}}}}_{t}$, and ${{{{\rm{RH}}}}}_{t}$ are temperature, rainfall, and relative humidity at week *t*, respectively; and ${{{{\rm{T}}}}}_{t-1}$, ${{{{\rm{R}}}}}_{t-1}$, and ${{{{\rm{RH}}}}}_{t-1}$ are lagged values of climatic variables from the previous week, *t*−1.

To capture short term temporal dependence for weekly cholera cases, we respecified the above model by including lagged outcome term representing the cholera cases in the preceding week (week *t*−1). The respecified model is as follows:

$$\log \left({\mu }_{t}\right)= 	 {\beta }_{0}+s\left({{Case}}_{t-1}\right)+s\left({{{{\rm{T}}}}}_{t}\right)+s\left({{{{\rm{R}}}}}_{t}\right)+s\left({{{{\rm{RH}}}}}_{t}\right)\\ 	 +s\left({{{{\rm{T}}}}}_{t-1}\right)+s\left({{{{\rm{R}}}}}_{t-1}\right)+s\left({{{{\rm{RH}}}}}_{t-1}\right)$$

Where, ${{Case}}_{t-1}$ denotes cholera cases in the preceding week, and the other variables same as described in the section above.

#### Yearly case data

To understand effect of the different factors on the yearly number of cholera cases a generalized negative binomial regression model was used. The general form of the implemented model is as follows:

$${{{\rm{E}}}}\left({Y}_{i}\right)={\mu }_{i}={e}^{{X}_{i}\beta }$$

Where, ${Y}_{i}$ is the total number of cholera cases in the *i*^th^ year; ${X}_{i}$ is the vector of predictor variables, and β is the vector of regression coefficients.

And the specific model is-

$$\log \left({\mu }_{i}\right)= \,	 {{\beta }_{0}}+{\beta }_{1}{({{nwT} > 25})}_{i}+{{\beta }_{2}}{({{nwR} > 100})}_{i}+{{\beta }_{3}}{(68{ < {nwRH} > 77})}_{i}\\ 	 +{{\beta }_{4}}{\left({VC}{{\_}}{Typing}1\right)}_{i}+{{\beta }_{5}}{\left({VC}{{\_}}{Typing}2\right)}_{i}$$

Where, ${({nwT} > 25)}_{i}$ is the number of weeks in the *i*th year with average weekly temperature exceeded 25 °C; ${({nwR} > 100)}_{i}$ is the number of weeks in the *i*th year with aggregated rainfalls exceeded 100 mm; ${(68 < {nwRH} > 77)}_{i}$ is the number of weeks in the *i*th year with average relative humidity within 68-77%; ${\left({VC\_Typing}1\right)}_{i}$ is the categorical variables referring the defined types of circulating *V. cholerae* O1 El Tor strains in the *i*^th^ year, it has two labels single dominant and multi-types; and ${\left({VC\_Typing}2\right)}_{i}$ is a categorical variable referring the observed evolutionary or transmission events of the circulating *V. cholerae* O1 El Tor strains in the *i*^th^ year, which have four labels including “same”, “changed proportion”, “Lineage added”, and “Linage dropped”. This model was designed based on the output of the first model (weekly data). Model with weekly data estimated favorable temperature, rainfall, and relative humidity for the increased number of cholera cases. Both models were estimated, and diagnosis analyses performed using R.

### Ethics statement

The Institutional Review Board (IRB) of icddr,b has approved the DDSS, its consent procedures, and data preservation and use policy. A separate IRB approval is not required for the use and analysis of anonymized DDSS data for research purposes^33^. In addition, this study analyzed genomic data collected under multiple IRB approved protocols at icddr,b, along with publicly available sequence data. No new samples or metadata were collected in this study. Therefore, a separate IRB was not required for this study.

## Results

### Dynamic changes in cholera cases in Dhaka city

**Fig. 1:**
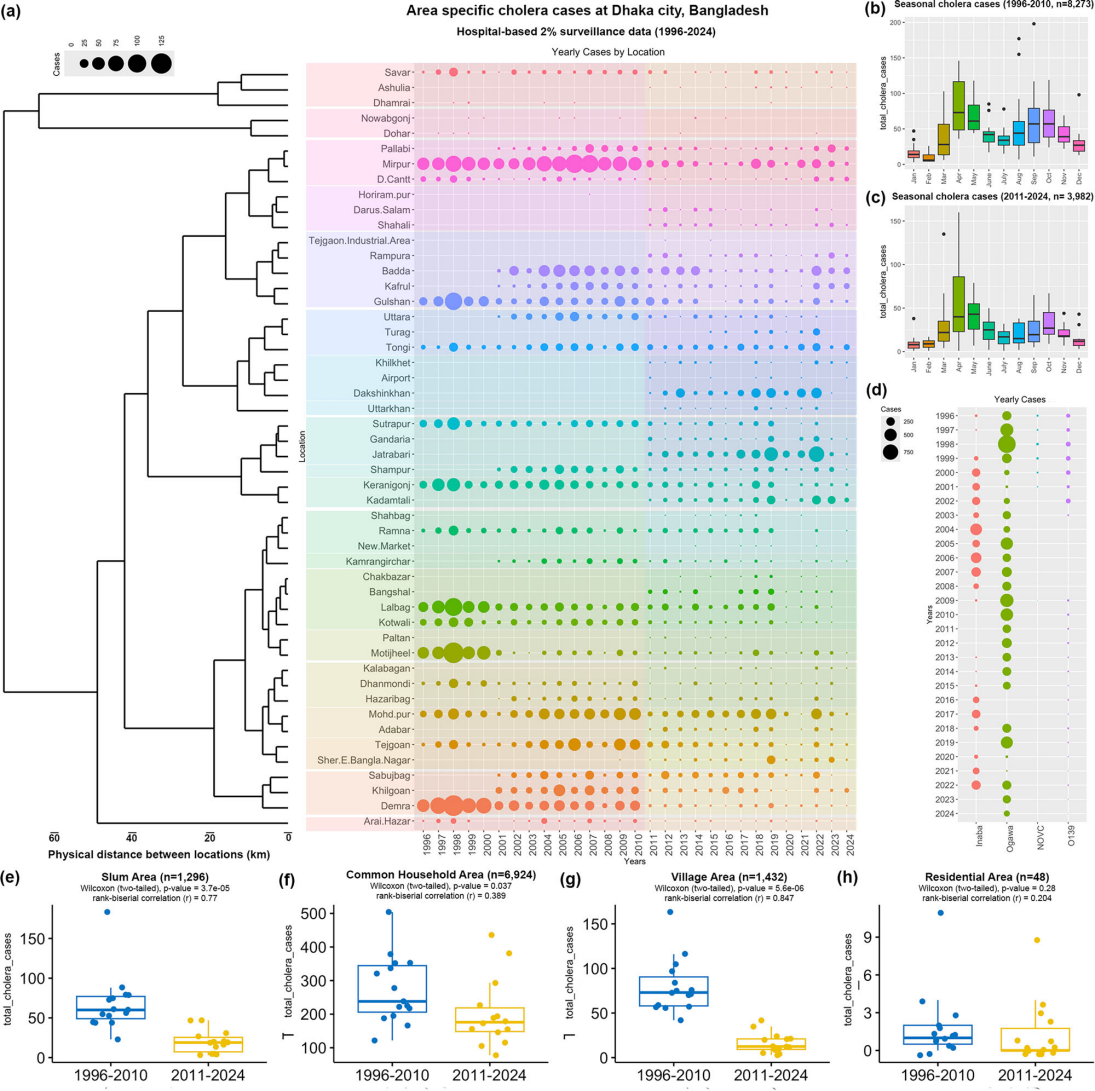
Transmission patterns, seasonality, and distribution of *V. cholerae* strains in Dhaka city 1996–2024. **a** shows geographical distance between different locations in Dhaka city, obtained from online sources, and hierarchical clustering allowed grouping of nearby locations. Yearly cholera case data for the locations were visualized, based on clustering structure. Box plots in the (**b**, **c**) show changes in the seasonal cholera patterns for the two periods (1996–2010 and 2011–2024). The total number of cholera positive patients enrolled under DDSS during 1996–2010 was 8273 and during 2011–2024 was 3982. **d** presents the distribution of *V. cholerae* strains by biotype and serotypes over the years. And (**e**–**h**) show a yearly total of cholera cases with respect to different types of households for the two periods, 1996–2010 and 2011–2024. Total number of cholera positive patients enrolled under DDSS during 1996–2024 varied across household types as follows: Slum (*n* = 1296), Common Household (*n* = 6924), Village (*n* = 1432), and Residential (*n* = 48). Error bars of box plots in the (**b**, **c**) and (**e**–**h**) indicate 95% confidence intervals around the median number of monthly and yearly cholera cases, respectively. Two-tailed Wilcoxon rank-sum test was used to test significant difference in number of cases between the year groups across household types (**e**–**h**), and rank bi-serial correlation was estimated as the effect size.

The cholera data obtained from 2% systematic hospital-based surveillance at Dhaka hospital of the International Centre for Diarrhoeal Disease Research, Bangladesh (icddr,b), from January 1996 to August 2024, were analyzed to map the cases and determining the transmission patterns of the disease in Dhaka city. The surveillance system enrolls every 50th patient admitted to the icddr,b hospital, Mohakhali, Dhaka, with appropriate consent^34^. These data showed that out of 76,815 patients screened 12,255 (16%) tested positive for cholera by laboratory culture or rapid diagnostic test (RDT). Results revealed that in Dhaka city recurrent endemic cholera continued year-round during the study period, 1996–2024, but the seasonal outbreaks of the disease occurred, forming a characteristic dual-peak pattern (Fig. 1a), once in March–May (pre-monsoon), and then in September–November (post-monsoon).

According to our case mapping data, cholera traced in wide areas of the mega city, Dhaka, but the disease incidence varied widely across different time points. The endemic cholera often turned into a large outbreak showing spatio-temporal shifts in the major hotspots of the disease (Figs. 1a, S1). Apart from the annual seasonal, and spatial variation in the disease incidence, we also observed temporal pattern of low and high cholera during the study period, 1996–2024. Of note, from Fig. 1, there was a dramatic decrease in the number of cholera cases observed in the well-known hotspots of the disease at Dhaka city, namely Mirpur, Mohammadpur, and Demra (Fig. 1a, 1996–2010, and 2011–2024, color shaded to show gradient); and this pattern of low-cholera began after 2010, and continued for several years. Consequently, post-2010 cholera occurred in significantly low number in different slums and common households of the Dhaka city (Fig. 1e-g).

Of the two defined peaks of the disease at Dhaka, the pre-monsoon (early) peak appeared to have significantly higher number of cases, as evident from the cholera surveillance data collected from 1996 to 2024. To understand if the observed shift in the cholera incidence at different areas of Dhaka city during the study periods, 1996–2010, and 2011–2024, had any impact on the seasonal dual-peak pattern, we plotted the confirmed cholera case data longitudinally across the above-mentioned time periods. Whilst the results revealed no apparent change in the seasonal dual-peak pattern of the disease, the overall number of cases reduced drastically after 2010, and cholera incidence was much lower in number for the following years (Fig. 1b-c). Also, during 2011–2024, the proportion of the overall seasonal disease incidence altered as the average number of cholera cases shifted mainly to the pre-monsoon season (34.4% to 42.8%), resulting in a significant reduction in the number of post-monsoon cases recorded in the past years, 1996–2010 (30.6% to 25.1%) (Supplementary note 1).

Despite reduction in the yearly average cholera cases recorded during the years 2011–2024, Dhaka city witnessed major upsurges of the disease in 2018, 2019, and 2022, with patients coming mainly from different cholera hotspots namely: Mirpur, Pallabi, Mohammadpur, Kadamtola, Jatrabari, Dakshinkhan, and Badda (Fig. 1a). In addition, the disease severity increased significantly in the recent years, compared to what was observed in 1996–2010 (Supplementary note 1). For example, the proportion of cholera patients with severe dehydration increased over time from 60.1% in 1996–2010 to 73% in 2011–2024 of the yearly confirmed cholera patients enrolled in the 2% surveillance system. In addition, patients having high stool frequency (16–20 times per day and >21 times per day) increased to 28.7% and 19%, respectively, compared to 13.4% and 9.4%, respectively, in the past period. Also, in recent upsurges of severe cholera, 55.7% of the patients seeking care at icddr,b hospital complained of abdominal pain, which was 46.6% for patients admitted in the preceding years, 1996–2010.

### Phenotypic and genetic changes in *V. cholerae* associated with endemic and epidemic cholera at Dhaka

**Fig. 2:**
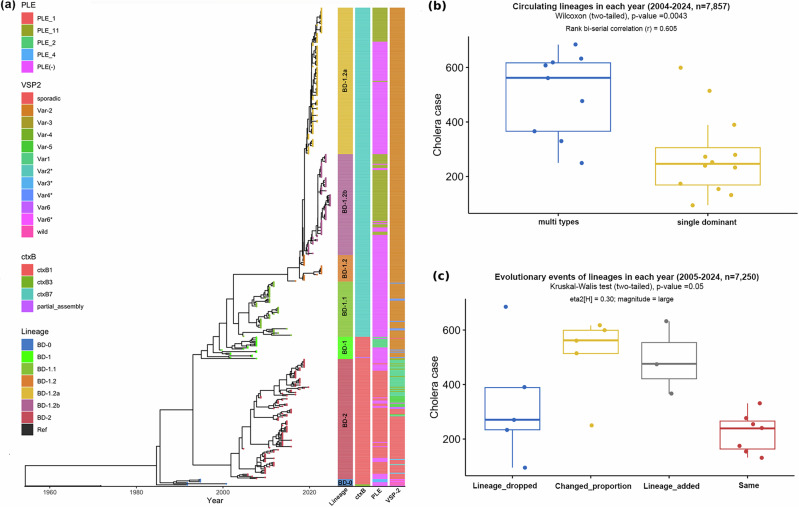
Lineage profiling and lineage association with cholera cases. **a** shows the maximum clade credibility tree visualized with year of isolation, defined lineages/subtypes, *ctxB* genotype, phage inducible chromosomal like elements (PLE), and vibrio seventh pandemic island-2 (VSP-2). Variants of VSP-2 were categorized based on the deletion patterns of genes (Supplementary Data 2), and variants of PLE were identified and described in recent studies^39,52^. **b** shows the relationships of single or multiple *V. cholerae* O1 El Tor lineages/sub lineages/genetic groups with the number of cholera cases between 2004 and 2024. A total of 7857 cholera positive patients were enrolled under DDSS during this period. Two-tailed wilcoxon rank-sum test was used to test significant difference between the year groups, and rank bi-serial correlation was estimated as the effect size. **c** shows the relationships of different evolutionary events, such as lineage drop (“Lineage dropped”), altered proportion of circulating lineages (“Changed proportion”), lineage newly introduced or re-emerged (“Lineage added”), and minimal change in current year from preceding year (“Same”), with the number of cholera cases between 2005 and 2024. A total of 7250 cholera cases were observed under the DDSS during this period.

We gathered the biotype and serotype-specific information, including the type of *V. cholerae* strains responsible for endemic and epidemic cholera, from routine 2% icddr,b Dhaka hospital-based surveillance between 1996 and 2024. Our data suggest temporal change in *V. cholerae* strains responsible for endemic and epidemic cholera in Dhaka city (Fig. 1d). *V. cholerae* etiology confirmed for the suspected cholera-like diarrhea between 1996 and 2024 included serogroups O1, O139, and non-O1/O139, while O1 El Tor predominated as the etiological agent for the ongoing seventh cholera pandemic at Dhaka. *V. cholerae* O139 and non-O1/O139 strains isolated from clinical cases of cholera-like diarrhea were limited in number and were insignificant as the etiological agent compared to O1 (Fig. 1a). Serological results revealed temporal changes in *V. cholerae* serotypes in cholera, and shifting from Inaba to Ogawa, and back to Inaba again, as endemic cholera progressed at Dhaka from 1996 to 2024. Although either of the two serotypes, Inaba or Ogawa, was predominantly associated with endemic and epidemic cholera in Dhaka city, the two serotypes overlapped and coexisted in varying proportions in cholera (Fig. 1d). The two serotypes were comparable in proportion in the massive cholera epidemic occurring in the 2022 when the Dhaka city was badly affected with a significant morbidity and mortality. Based on our data, the Ogawa serotype strains were found predominantly associated with cholera in 2023–2024 (Fig. 1d). To gain a genomic view of these changes we included the whole genome sequence data for *V. cholerae* O1 El Tor strains associated with cholera in Dhaka between 1991 and 2024 (Supplementary Data 1). Both maximum likelihood based phylogeny and maximum clade credibility tree results revealed that the BD−1.2, which we found by analyzing a limited number of sequenced *V. cholerae* strains (*n* = 21) isolated from the 2022 massive cholera epidemic at Dhaka (Monir et al., 2023^13^), also was accompanied by two other subtypes: BD-1.2a and BD-1.2b, as we included more genomic data with our current analysis in this study (Figs. 2a, S2). Nonetheless, BD-1.2b expanded and was predominant as the etiological agent of cholera in 2023–2024, and the BD−1.2 and BD−1.2a subgroup strains were not found associated with cholera in Dhaka since 2023 (Fig. 2a, Supplementary Data 1).

Cholera genome sequence data for each year following 2004 assisted investigation of transmission dynamics of the lineages over the years, until 2024 (Table 1). Based on the identified lineages and their prevalence, two typing schemes were used to categorize transmission and genomic evolutionary events of the *V. cholerae* O1 El Tor strains responsible for the currently ongoing 7th cholera pandemic in Bangladesh. Of these, VC_typing1: “Multi types” indicates multiple sub-lineages of *V. cholerae* El Tor strains present in high proportions; and the “Single dominant” indicates one lineage predominant ( > 80%), with others <20% or absent, as the etiological agent of cholera. For example, in 2012, *V. cholerae* El Tor strains belonging to lineages BD−1.1 and BD-2 were present at 47% and 53% prevalence, respectively. The multiple lineage strains were co-present at a high proportion in 2012. However, in 2013, BD-2 lineage strains were predominant, reflecting single lineage dominance in cholera transmission at Dhaka. Our genome analysis data showed a strong association (Wilcoxon test, *p* = 0.0043, rank bi-serial correlation = 0.605) of the multi-type lineage transmission with a significantly higher number of cholera cases at Dhaka city (Table 1, Fig. 2b).

**Table 1:** *Vibrio cholerae* O1 El Tor associated cholera cases and lineage presence

Year	Cholera case	Lineages	Proportions (%)	VC_typing1	Description (VC_typing1)	VC_typing2	Description (VC_typing2)
2004	607	BD−1, BD−1.1, BD-2	33,33,33	Multi types	Different types strains with similar proportion	Unknown	Sequence data for 2003 is not available
2005	684	BD−1, BD-2	50,50	Multi types	Different types strains with similar proportion	Lineage dropped	BD-1.1 strains were not identified, and changed proportion for other two lineages
2006	617	BD-1, BD-2	66,33	Multi types	One dominates but other presents in high proportion ( > 20%)	Changed proportion	Changed proportion than previous year
2007	632	BD-1, BD-1.1, BD-2	70,24,6	Multi types	One dominates but other presents in high proportion ( > 20%)	Lineage added	Changed proportion than previous year and BD-1.1 strains identified with substantial proportion
2008	389	BD-1.1, BD-2	82,18	Single dominant	Single lineage dominates ( > 80%)	Lineage dropped	Changed proportion than previous year, and BD-1 strains were not identified
2009	599	BD-1.1, BD-2	7,93	Single dominant	Single lineage dominates ( > 80%)	Changed proportion	Changed proportion than previous year
2010	514	BD-1.1, BD-2	86,14	Single dominant	Single lineage dominates ( > 80%)	Changed proportion	Changed proportion than previous year
2011	250	BD-1.1, BD-2	56,44	Multi types	Different types strains with similar proportion	Changed proportion	Changed proportion than previous year
2012	331	BD-1.1, BD-2	47,53	Multi types	Different types strains with similar proportion	Same	Similar proportion as previous year
2013	271	BD-2	100	Single dominant	Single lineage dominates ( > 80%)	Lineage dropped	Changed proportion than previous year, and BD-1.1 strains were not identified
2014	278	BD-1.1, BD-2	6,94	Single dominant	Single lineage dominates ( > 80%)	Same	Similar proportion as previous year
2015	239	BD-1.1, BD-2	11,89	Single dominant	Single lineage dominates ( > 80%)	Same	Similar proportion as previous year
2016	153	BD-1.2, BD-2	8,97	Single dominant	Single lineage dominates ( > 80%)	Same	Similar proportion as previous year, and BD-1.2 strains identified with negligible proportion
2017	254	BD-2	100	Single dominant	Single lineage dominates ( > 80%)	Same	Similar proportion as previous year
2018	366	BD-1.2, BD-2	63,37	Multi types	One dominates but other presents in high proportion ( > 20%)	Lineage added	Changed proportion than previous year and BD-1.2a strains identified with substantial proportion
2019	476	BD-1.2a, BD-1.2b, BD-2	55,41,4	Multi types	Different types strains with similar proportion	Lineage added	Changed proportion than previous year, BD-1.2a and BD-1.2b strains identified with substantial proportion
2020	95	BD-1.2a, BD-1.2b	86,14	Single dominant	Single lineage dominates ( > 80%)	Lineage dropped	Changed proportion than previous year, and BD-2 strains were not identified
2021	174	BD-1.2, BD-1.2a, BD-1.2b	1,93,6	Single dominant	Single lineage dominates ( > 80%)	Same	Similar proportion as previous year, and BD-1.2 strains identified with negligible proportion
2022	562	BD-1.2, BD-1.2a, BD-1.2b	12,35,52	Multi types	Different types strains with similar proportion	Changed proportion	Changed proportion than previous year and BD-1.2 strains identified with substantial proportion
2023	234	BD-1.2b	100	Single dominant	Single lineage dominates ( > 80%)	Lineage dropped	Changed proportion than previous year, and BD-1.2b strains were not identified
2024	132	BD-1.2b	100	Single dominant	Single lineage dominates ( > 80%)	Same	Similar proportion as previous year

In the analysis, cholera cases refer to confirmed disease caused by *V. cholerae*, identified in the 2% hospital-based surveillance over multiple years. Lineages represent circulating *V. cholerae* O1 El Tor strains isolated in Bangladesh, with proportions determined from the sequence data (Supplementary Data 1).

Also, the association of genomic evolutionary events, such as lineage drop (“Lineage dropped”), change in proportion of the circulating lineages in current year than the preceding year (“Changed proportion”), lineage newly introduced or re-emerged (“Lineage added”), and minimal change in the current year from the preceding year (“Same”) with the number of confirmed cholera cases were investigated. VC_typing2 was introduced by including evolutionary events (Table 1). Non-parametric Kruskal–Wallis rank sum test showed significant (*p* = 0.05, eta2[H] = 0.30) association of the number of cholera cases with the *V. cholerae* genomic evolutionary events. During the years of significant changes in the circulating lineage proportion or lineage added in the year, the number of cholera cases increased significantly, suggesting that *V. cholerae* genomic evolutionary events and the dynamics of outbreak strains can play a significant role in shaping the epidemiology of cholera at Dhaka city.

### Cholera case data modelling incorporating climate and genetic factors

**Fig. 3:**
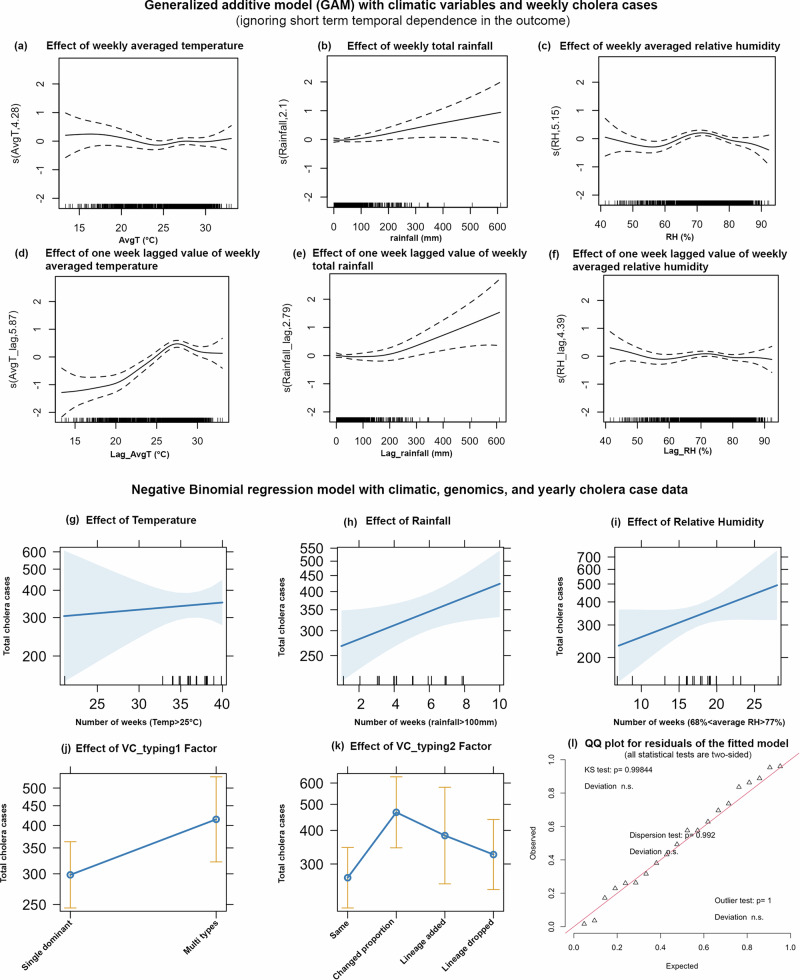
Effect plots of various factors on weekly and yearly incidence of cholera. Here, **a**–**f** show the effects plots based on GAM model for weekly cholera (model ignoring short term temporal dependence in the outcome), y-axis = estimated effect of smooth function of predictor on outcome (log of cholera cases) and x-axis = predictor variables. Solid lines show the estimated smooth effects, while dashed line indicate 95% confidence interval. If the smooth function (solid line) approximates zero and within the shaded region (95% confidence interval), the predictor likely has no significant effect. Solid line above or below zero with narrow confidence interval suggests significant effect. And, figures **g**–**k** show effect plots based on the negative binomial regression model for yearly cholera case data. For the (**g**–**i**), the solid blue lines refer model-predicted total number of cholera cases, and the shaded bands indicate 95% confidence interval around the predicted values. For the (**j**, **k**), the points show estimated means from the model for each factor level, with error bars representing 95% confidence intervals. **l** shows the goodness of fit of the model based on residual analysis. Uniformity of residual was evaluated using two-sided Kolmogorov–Smirnov test, and simulation-based dispersion and outliers tests using DHARMa R-package.

Climate factors, including temperature and rainfall, have been shown to be associated with cholera cases in Dhaka^35–38^. In this study, climatic data, including temperature, rainfall, and relative humidity for Dhaka, collected by the Bangladesh Meteorological Department, were included in the cholera models employing hospital-based surveillance studies by the icddr,b hospital, Dhaka. Generalized additive model (GAM) was used to fit climate data (average temperature, average relative humidity, and total rainfall) for the current and preceding (lagged) weeks with the weekly total of cholera cases. The climate data for the preceding week were included to estimate the delayed effects. This analysis showed a significant association of the weekly total cholera cases with the weekly average relative humidity (*p*-value < 2e-16), preceding week average temperature (*p*-value < 2e-16), and preceding week total rainfall (*p*-value = 0.0236) (Supplementary Data 3). Results of the GAM were plotted with each panel representing the effect of an individual term included in the model (Fig. 3a-f). Results indicate temperature and rainfall from the preceding week, and current relative humidity significantly predicted case counts, and the number of cholera cases increased when the preceding week average temperature was above 25 °c, the preceding week total rainfall was greater than 100 mm, and the weekly average relative humidity ranged from 68 to 78%.

Although, the fitted GAM yielded significant association for the climate variables, we detected a substantial positive autocorrelation in the residuals, indicating that weekly cholera counts were not independent over time (Fig. S4a). This temporal dependence is epidemiologically meaningful, as high case counts in one week are likely to be followed by high counts in subsequent weeks due to (i) ongoing transmission dynamics, and (ii) persistence of environmental contamination in the hotspots.

To address this issue, we re-specified the model by including cholera cases in the previous week (week *t* − 1), which explicitly accounts for a short-term temporal dependence in the outcome. Analysis showed the respecified model with the lagged term effectively eliminated autocorrelation (Fig. S4b). In addition, model fit and explanatory power improved substantially, with a marked reduction in AIC (without week *t* − 1 outcome in the model: 9076.321; and with week *t* − 1 outcome in the model: 7822.365) and a notable increase in adjusted R² (without week *t* − 1 outcome in the model: 0.167; and with week *t* − 1 outcome in the model: 0.724) (Supplementary Data 3). It is also noted that although inclusion of lagged outcome solves the autocorrelation problem, it reduces statistical power for “relative humidity” (Supplementary Data 3, Fig. S5). However, statistical significance for average temperature and rainfall remained unchanged. These results suggest probable correlation of relative humidity with transmission persistence of cholera during an outbreak.

Dhaka is a prominent focus, a hotspot for endemic cholera in Bangladesh with cases occurring throughout the entire year, but with seasonal and annual variation. Based on the analysis results of generalized additive models, it is expected that the total annual number of cholera cases was higher in years with more weeks (duration) with an average temperature >25 °C, total rainfall >100 mm, and relative humidity between 68 and 78%. To evaluate this, we introduced new variables into our model, including the number of weeks with average temperature above 25 °C, the number of weeks with total rainfall above 100 mm, and the number of weeks with relative humidity between 68 and 78%. A significant association was found between the genetic variation and the transmission events of *V. cholerae* O1 El Tor strains with the yearly incidence of cholera cases (Fig. 2b-c), further validated by negative binomial regression analysis (Fig. 3g-l).

Model diagnostic analyses, including AIC evaluation, likelihood ratio tests (LRT), and QQ plots for residuals, were applied (Fig. 3l*,* Supplementary Data 4); and all suggest a good fit for the model with the data. According to the fitted model, the yearly total number of cholera cases were significantly associated with the number of weeks with total rainfall exceeding 100 mm (*p*-value = 0.0199), number of weeks with average weekly relative humidity between 68 and 78% (*p*-value = 0.0507), multiple genetic types of *V. cholerae* O1 El Tor strains (*p*-value = 0.0444), and a change in proportion of the lineages than that existing in the preceding year (*p*-value = 0.0036). Although temperature was associated with weekly cases, the number of weeks with high temperature was not associated significantly with total yearly cases, and thus, temperature cannot explain the yearly variation in the number of cholera cases. In this analysis, we modeled the number of weeks with high temperature, a metric that shows limited interannual variability (~34–40 weeks per year). This restricted variability substantially reduces statistical power to detect an association with total annual case counts. Nonetheless, in aggregate, the rainfall, humidity, and evolutionary and transmission events appear crucial for cholera epidemiology, and are concluded to be significantly associated with the interannual variations of the overall (high or low) number of cholera cases observed over the years in Dhaka city.

### Recent changes in *V. cholerae* O1 El Tor genome

Previously, we presented data of the comparative genomics analysis of *V. cholerae* El Tor lineages BD-1, BD-1.1, BD-1.2, and BD-2 associated with endemic and epidemic cholera in Bangladesh^3,13^. Here, we classify two additional BD-1.2 sub-clade strains designated BD-1.2a and BD-1.2b. Comparative genomic analysis results showed that all 42 BD-1.2b genomes isolated from cholera between 2023 and 2024 carried a new type of phage inducible chromosomal island-like element (PLE11) presumably providing adaptive advantage^39^. Variant of VSP-2 remained unchanged (Var-4) for BD-1.2b, which is specific to the strains similar to BD-1 lineage (Supplementary Data 2). Association analysis of the genetic variants with all of the defined lineages of *V. cholerae* O1 El Tor strains using Fisher exact test identified 208 single nucleotide variants (SNVs) that were significantly associated (*P*-value < 0.02) of which 17 SNVs were unique for BD-1.2b (Supplementary Data 5). These unique genetic variants were detected in 63-100% of BD-1.2b genomes, of which 10 were missense SNPs, 4 synonymous SNPs, 2 intergenic SNPs, and one deletion mutant. The SNVs that fell within the coding regions of BD-1.2b genes are predicted to encode a methyl-accepting chemotaxis protein (VC_RS17940), histone deacetylase enzyme (VC_RS09835), HPT domain-containing protein (VC_RS09805), PTS ascorbate-specific subunit IIBC (VC_RS14510), bifunctional proline dehydrogenase/L-glutamate gamma-semialdehyde dehydrogenase (*putA*) (VC_RS18400), enoyl-ACP reductase *fabV* (VC_RS17100), phenylalanine 4-monooxygenase (VC_RS17290), amino acid ABC transporter permease (VC_RS18240), TRAP transporter permease (VC_RS14055), autotransporter assembly complex family protein (VC_RS12285), diaminopimelate epimerase (VC_RS00585), glycine C-acetyltransferase (VC_RS17550), and zinc ABC transporter ATP-binding protein *znuC* (VC_RS10025). Combined, these genes may serve for the virulence of the bacterium by providing important fitness advantage to adapt under the changing environment and flourish through nutrient acquisition and maintenance of metabolism and cellular structure under stress.

To understand the functional importance of the unique mutations found in the BD-1.2b strains associated with endemic and epidemic cholera, we analyzed the genomic data employing multiple tools namely- BLOSUMS62^28,40^, Grantham’s scores^29^, SIFT^30^, and DDMut^31^. (Table 2). While the negative values of the BLOSUM62 score indicated non-random substitutions of bases and potential functional consequences, higher Grantham’s distance inferred radical changes in amino acid properties and non-conservative nature of the mutations which might contribute to increased adaptation, including functional divergence. Based on our results, the acquired unique mutations in BD-1.2b were mostly non-conservative and had a functional potential according to both BLOSUM62 scores and Grantham’s Distance. In addition, DDMut and SIFT web server suggested the acquired mutation impacted protein stability and function, respectively, and thus, contribute to survival and other functional competencies of the bacterium.

**Table 2:** Summary of the missense mutation analysis of BD-1.2b

LOCUS_TAG	PRODUCT	Mutation	BLOSUM62	Grantham’s Distance	SIFT	DDMut
VC_RS17940	Methyl-accepting chemotaxis protein	I161S	−2	142	0 (AFFECT PROTEIN FUNCTION)	−1.85 kcal/mol (Destabilising)
VC_RS09805	Hpt domain-containing protein	G277V	−3	109	0.01 (AFFECT PROTEIN FUNCTION)	–0.01 kcal/mol (Destabilising)
VC_RS14510	PTS ascorbate-specific subunit IIBC	I354T	−1	89	0 (AFFECT PROTEIN FUNCTION)	–2.11 kcal/mol (Destabilising)
VC_RS18400	Bifunctional proline dehydrogenase/L-glutamate gamma-semialdehyde dehydrogenase PutA	A600V	0	64	0.02 (AFFECT PROTEIN FUNCTION)	−1.17 kcal/mol (Destabilising)
VC_RS17100	Enoyl-ACP reductase FabV	P149H	−2	77	0 (AFFECT PROTEIN FUNCTION)	–0.15 kcal/mol (Destabilising)
VC_RS18240	Amino acid ABC transporter permease	P191S	–1	74	0 (AFFECT PROTEIN FUNCTION)	−0.46 kcal/mol (Destabilising)
VC_RS17290	Phenylalanine 4-monooxygenase	Q19L	−2	113	0.26 (TOLERATED)	0.22 kcal/mol (stabilising)
VC_RS14055	TRAP transporter permease	S241A	1	99	0.02 (AFFECT PROTEIN FUNCTION)	0.21 kcal/mol (stabilising)
VC_RS12285	Autotransporter assembly complex family protein	T266I	−1	89	0 (AFFECT PROTEIN FUNCTION)	0.19 kcal/mol (stabilising)

In BLOSUM62 (ranges from −4 to 11), negative values indicate substitutions that are rarely observed in evolution (i.e., non-conservative mutation) while positive values indicate commonly observed mutations in evolution (i.e., conservative mutation).

In Grantham’s distance (range from 5 to 215), higher score indicates significant change in amino acid properties.

A SIFT score of less than 0.05 indicates that the mutation is likely to affect protein function, while a score greater than 0.05 suggests that the mutation is “tolerated” and has a minimal impact on protein function.

In DDMut, Negative ΔΔG values indicated destabilizing mutations, while positive ΔΔG values suggested stabilizing mutations.

## Discussion

Cholera, an old scourge, continues unabated, killing people and affecting millions despite the goal set forth by the Global Task Force for Cholera Control (GTFCC) to reduce cholera mortality by 90% and eradicate transmission of the disease in at least 20 countries by 2030^41^. However, many endemic countries have witnessed remarkable case surges of cholera in recent years. According to the World Health Organization (WHO), forty-four countries have reported cholera in 2022, with 25% increase from 35 countries as compared to 2021^42^. Of note was the massive epidemic that struck Bangladesh in 2022, and the icddr,b Dhaka hospital was overwhelmed with well over 1300 patients per day, a number that surpassed all past records of the daily patients^43^. Here we present integrated analyses results of the *V. cholerae* whole genome sequence data, the natural climate factors such as temperature, rainfall, and humidity, and cholera case data of the world’s largest DDSS to elucidate the cholera transmission dynamics, especially how these factors contribute to the epidemiology of the disease, including the massive upsurges like the one occurring at Dhaka city in 2022. This study is the first to reflect the intricate link of *V. cholerae* genomic evolutionary dynamics and the climate factors with the epidemiology and disease incidence, highlighting the importance of genomic surveillance in predicting epidemic cholera and designing intervention and preventive strategies against the disease showing massive upsurges recently.

Despite the etiology of endemic and epidemic cholera, *V. cholerae*, is toxigenic serogroup O1 biotype El Tor, there is no known bacterial factor(s) that could explain the observed decrease in the overall number of cases in the key cholera hotspots of the Dhaka city after 2010 (Fig. 1a, 1996–2010 and 2011–2024 color shaded to show gradient). The presumed major contributing factor for the sharp decline of the disease in Mirpur could be the impact of cholera vaccination in this area by an ICVB campaign that administered two doses to 123,661 individuals and a single dose to 18,178 people during March 2011^44,45^. Although the low cholera observed in other unvaccinated areas of the Dhaka city remains unexplained, this may be due to potential interconnections and vaccine-induced reduction in cholera transmission in the neighboring area of the city. The other potential factor(s) may be the “Turnaround Plan” undertaken in 2010 by the Dhaka Water Supply and Sewerage Authority (DWASA) with aid from the Asian Development Bank and other partners, to implement the safe water supply for the city dwellers by connecting slum areas to improved water^46^. The DWASA initiatives to ensure safe drinking water in slum areas, thus, appear crucial for the increasing slum dwellers in Dhaka city, as they often suffer from cholera-like symptoms [“we have a permanent booking at the cholera hospital (icddr,b)” says Aisha Aktar, a resident of the Shattola (7-story) slum]^46^. In addition, surveillance data showed a significant improvement of the living conditions and hygiene (e.g. Toilet facility) status of the high-risk population of the Dhaka city (Supplementary note 1). For examples, houses with cement floors rose from 59.7% to 89.3%, and brick walls from 44.7% to 78.3%, and tin roof to concrete roofs (43.3%) became the most common in 2011–2024, supporting the supposition of hygiene improvement.

The relationship of climate factors and cholera has been well-established, although the seasonal dual peak of the disease at Dhaka city remains an enigma. Several studies have shown linkage of the pre-monsoon early peak of the year with the rising summer temperature in March and April, while the post-monsoon peak to flooding, and the resulting contamination of the city water supplies^36^.

In Bangladesh, the dramatic increase in cholera cases following the several consecutive years of low cholera was marked with subsequent large cholera outbreaks in the recent past years. Our comparative genomic analysis data of *V. cholerae* strains associated with the 2022 epidemic provided important insights reflecting temporal predominance of a particular El Tor lineage, introduction and reemergence of lineage, and serotype switching events in the massive outbreak of cholera at Dhaka^3,13^. Here we present further data showing that the genomic evolutionary changes in *V. cholerae* El Tor were associated significantly with the extent of the disease epidemiology in respect to severity and transmission in Dhaka city. The genome analysis data presented in this study provide overwhelming evidence of a significant association (Wilcoxon test, *p* = 0.0043) of the interannual low and high cholera with the transmission of a single lineage and multiple sub-lineages of *V. cholerae* El Tor, respectively, in Dhaka city.

In this study, the combined results of the analysis of *V. cholerae* genomic evolutionary factors, routine cholera surveillance, and climate related data provide direct evidence of their inter-relationship in shaping the epidemiology and transmission of the disease in Dhaka city. Our prospective analysis to infer the genomic changes in *V. cholerae* El Tor associated with cholera in Dhaka showed expansion of the previously reported subgroup of BD-1.2 lineage (Monir et al., 2023), designated BD-1.2b, while none of the of other subgroup strains of the lineage, namely BD-1.2a, were identified from among the cholera outbreak strains isolated in 2023–2024, suggesting selective advantage for the BD-1.2b strains. Our comparative genomic analysis data showed BD-1.2b strains isolated in 2023 and 2024 carried a phage inducible chromosomal island like elements (PLE11), which, presumably, provided selective advantage for the sublineage strains to overcome adverse environmental conditions and flourish, evading specific lytic bacteriophage attack^39^. The selective advantage for the BD-1.2b strains might also lie with the observed mutations in genes encoding proteins and enzymes involved in crucial functions related to environmental adaptation and proliferation by subsiding the other subtypes of the bacterium. For example, the methyl-accepting chemotaxis proteins (MCPs) are the transmembrane receptors that assist cells in adapting to environmental changes by adjusting their methylation state in response to chemical stimuli^47^. Likewise, the Enoyl-ACP reductase (EC 1.3.1.9) (ENR) is involved in fatty acid biosynthesis, which is crucial because it produces building blocks for lipids, essential components of cell membranes, energy storage molecules (triglycerides), and signaling molecules, vital for cellular structure, function, and energy metabolism^48^. In addition, the L-ascorbate phosphotransferase system (PTS)^49^ allows efficient transport and utilization of vitamin C (L-ascorbate) as a carbon source by actively bringing it into the cell while simultaneously phosphorylating it, a key step in metabolism, providing an energy advantage for the bacteria^50^. The overall data presented in this study suggest the unique mutations in BD-1.2b strains might provide selective advantage for the bacterium to evolve by adapting to the dynamic conditions in the aquatic environments and the host intestine.

Previous studies have described various climate factors, including temperature and rainfall, and their influence on the incidence of cholera^14,15,32^. One of the most intriguing findings of the present study may be the consensus climatic factors found associated with cholera cases from the hospital-based surveillance data gathered at icddr,b, Dhaka hospital using a generalized additive model. The temperature, rainfall, and relative humidity were found to be linked with the seasonal variation in the number of cholera cases in Dhaka city. In our study, 1-week lagged weekly averaged temperature >25 °C, 1-week lagged weekly total rainfall > 100 mm, and weekly average relative humidity ranging from 68% to 72% were associated positively with the increasing number of cholera cases. Insignificant results of the humidity effect after accounting for short-term dependence in the outcome (cholera cases) suggest that its association may operate indirectly through persistence of transmission rather than exerting an independent short-term effect. These data reinforce findings of the earlier studies showing a delayed effect of the rainfall^14^ and temperature^35^ on the increasing number of cholera cases in Dhaka city, and also, investigations that successfully linked seasonal temperature and related climate factors with cholera cases in Bangladesh^32^.

Dhaka city is an important hotspot for cholera in Bangladesh, where endemic disease often turns into large epidemic, resulting high morbidity and mortality. Although recurrent cholera occurs seasonally, the number of cholera positive cases varies temporally (Fig. 1a-h). To understand the interannual variation in the number of cholera cases, we developed a model by incorporating climate and *V. cholerae* genomic data, and employing negative binomial regression. Our model diagnostic analysis ensured reliability of the fitted model and estimated parameters to obtain a robust outcome. The data indicate that in those years with a higher number of weeks having climatic factors favorable for cholera, a larger number of cholera cases were recorded. In addition, genomic evolutionary events were also found to have a significant association with the yearly variations in the number of cholera cases. For example, in high cholera, as observed in 2022 ^13^, multiple subtypes of the El Tor sub-lineage strains were isolated, with the proportion of circulating lineages different from those existing in the preceding year, 2021, but also with the 28 weeks (maximum of 21 years analyzed, 2004–2024) of relative humidity that can favorably support cholera incidence in Dhaka city. This study thus, provides evidence that the large cholera outbreaks, namely the one causing havoc at Dhaka city in 2022, was an aggregative result of *V. cholerae* bacterial genomic, climatic (ecological), and as yet unidentified human (biological) and other factors, reflecting the very complex and interdisciplinary nature of the epidemiology and transmission of cholera. It is thus prudent that *V. cholerae* genomic surveillance, together with the routine monitoring of climate (environmental) and host (human) factors, appears crucial for predicting epidemics and designing management and preventive interventions against the deadly disease, cholera, in Bangladesh.

## Supplementary information

Supplementary Information

Description of Additional Supplementary Files

Data S1-S8

## Data Availability

To obtain individual-level DDSS data, interested researchers may contact with the Head of Research Administration at icddr,b (https://www.icddrb.org/senior-leadership-team). Climatic data used in this study can be obtained from the Bangladesh Meteorological Department. Interested researchers may request access through online portal (https://live8.bmd.gov.bd). The accession IDs of the sequenced *V. cholerae* O1 El Tor strains used in this study are provided in Supplementary Data 1. All other relevant information and source data are provided with this article, in Supplementary Data and in the figshare database^51^.

## Code availability

The source codes for the performed analyses for this study are available on GitHub (https://github.com/mamunmonir/Climate_Vibrio_Epidemiology), and also in the figshare database^51^. Details of the specific version of other software used in this study are provided in the method section.
