# Supplementary material for: Genomic evolution and climate related drivers of cholera surges in Dhaka Bangladesh between 1996 and 2024
Source: Commun Med (Lond). 2026 May 22;6:440. doi: 10.1038/s43856-026-01620-8 (PMC13469620; doi:10.1038/s43856-026-01620-8)
Supplement: Supplementary file 1 [file 43856_2026_1620_MOESM1_ESM.pdf]

Genomic Evolution and Climate Related Drivers of Cholera Surges in Dhaka Bangladesh between 1996 and 2024

Supplementary Information

AUTHORS

Md Mamun Monir[1], Khalilur Rahman[1], Mohammad Tarequl Islam[1], Marzia Sultana[1], Wali Ullah[1], Sardar Muhammad Osail[1], Md Alimul Islam Ridoy[1], Abdullah Al-Mamun[1], Shirajum Monira[1], Kimberley D. Seed[2], Andrew Camilli[3], Carla Mavian[4], Nicholas Thomson[5,6], Rita R. Colwell[7,8], ASG Faruque[1], Tahmeed Ahmed[1], and Munirul Alam[1]

*Correspondence to: Munirul Alam (munirul@icddrb.org)

AFFILIATIONS

[1]icddr,b, International Centre for Diarrhoeal Disease Research, Bangladesh, Dhaka, Bangladesh
[2]Department of Plant and Microbial Biology, University of California, Berkeley, Berkeley, CA 94720, USA
[3]Department Molecular Biology and Microbiology, Tufts University, 136 Harrison Avenue, Boston, MA 02111, USA
[4]Emerging Pathogens Institute, Department of Pathology, College of Medicine, University of Florida, Florida, 32601, USA
[5]Parasites and Microbes Program, Wellcome Sanger Institute, Wellcome Genome Campus, Hinxton, Cambridge CB10 1SA, UK
[6]London School of Hygiene and Tropical Medicine, London, UK
[7]Cell Biology and Molecular Genetics, University of Maryland, College Park, MD, USA
[8]University of Maryland Institute for Advanced Computer Studies, College Park, MD, USA

Supplementary note 1:

Yearly cholera cases and seasonal variation (1996-2010 and 2011-2024)

In Figure 1a, we observed two different patterns in the annual number of cases before and after 2010. To investigate details, we tabulated the total number of cases (median, minimum, and maximum) across two periods: 1996–2010 and 2011–2024 (**Supplementary Data 6**). A substantial decline was observed in the later period 2011–2024, the median of annual confirmed cholera cases significantly dropped from 536 (range: 284-1109) to 265.5 (range: 104-575)

(Wilcoxon rank-sum test, $p<0.001$). Seasonal cases (Pre-monsoon, and post-monsoon) was also significantly declined ($\chi^2=85.68$, $p<0.001$) during 2011-2024 on average (**Supplementary Data 7**). Majority of yearly cholera in 2011-2024 period occurred in pre-monsoon (42.8%) and 25.1% of yearly cholera cases occurred during the pre-monsoon (March-May) and post-monsoon (September-November) seasons, respectively, whereas during these seasonal periods in the 1996-2010 were 34.4% and 30.6%, respectively. These results suggest significant reduction of the percentage of post-monsoon cholera in Dhaka in the recent years.

Participant characteristics of the cholera patients

Age, sex, and father education: The distributions of cholera cases among different age groups (infants, children, adults, older adults) changed significantly ($\chi^2=329.81$, $p<0.001$) between the two time periods (**Supplementary Data 8**). Adults remained the most affected group, with their case proportion increasing from 48.4% in the 1996-2010 period to 61.7% in the 2011-2024 period, while the proportions of infant (25.9% to 16.4%) and child (17.3% to 10%) cases declined. In both periods, males were more affected than females (1996-2010: Male 55.2% and Female 44.8%; 2011-2024: Male 59.8% and Female 40.2%). The father's education showed a significant association with cholera risk. In 1996-2010, 64.4% of cases had no parental education, while this proportion dropped to 56.9% in 2011-2014. Interestingly, the percentage of cholera cases whose fathers had some formal education (especially 4-5 years) increased in 2011-2024.

Patients living conditions: Common households accounted for most of the cholera cases in both periods. Significant improvements were observed in housing materials and infrastructure, but cholera cases increased substantially among cemented floors from 59.7% in 1996-2010 to 89.3% in the 2011-2024 period. The household wall ($\chi^2=2509.03$, $p<0.001$) and roof type ($\chi^2=4350.06$, $p<0.001$) differed significantly among cholera cases between the periods. In 1996-2010, cholera cases were most common among brick walls, followed by bamboo (22.2%), and corrugated tin (18.1%). In 2011-2024, brick walls become overwhelmingly dominant (78.3%), declining in other materials. The majority of cholera cases were from homes with corrugated tin roofs (63.4%) and concrete roofs (19.8%) in 1996-2010, however, concrete roofs became the most common (43.3%),

and corrugated tin dropped to 4.2% in 2011-2024. Cholera cases were primarily associated with semi-sanitary (55.2%), dughole (23.3%), and open pit (11.8%) toilets in 1996-2010. In contrast, semi-sanitary toilets accounted for the vast majority of cases (88.4%) in 2011-2024, no-fixed place, and hanging toilets became rare in recent times.

Clinical symptoms: Regarding the clinical symptoms of cholera, dehydration, number of stools, vomiting, and abdominal pain among cholera cases differed significantly (p-value < 0.001) in both periods, but stool character (p-value=0.466), mucus or blood in stool (p-value=0.206) among cholera cases were independent of time period. Most of the patients had no visible mucus in their stools during both periods. The proportion of patients with severe dehydration increased from 60.1% in 1996-2010 to 73% in the 2011-2024 period. Additionally, 28.7% cholera cases experienced 16-20 stools per day, and 19% experienced more than 21 stools per day, compared to 13.4% and 9.4% respectively, during 1996-2010. Moreover, 55.7% of cases reported abdominal pain in 2011-2024, up from 46.6% in the earlier period, indicating a rise in severe clinical symptoms in cholera patients.

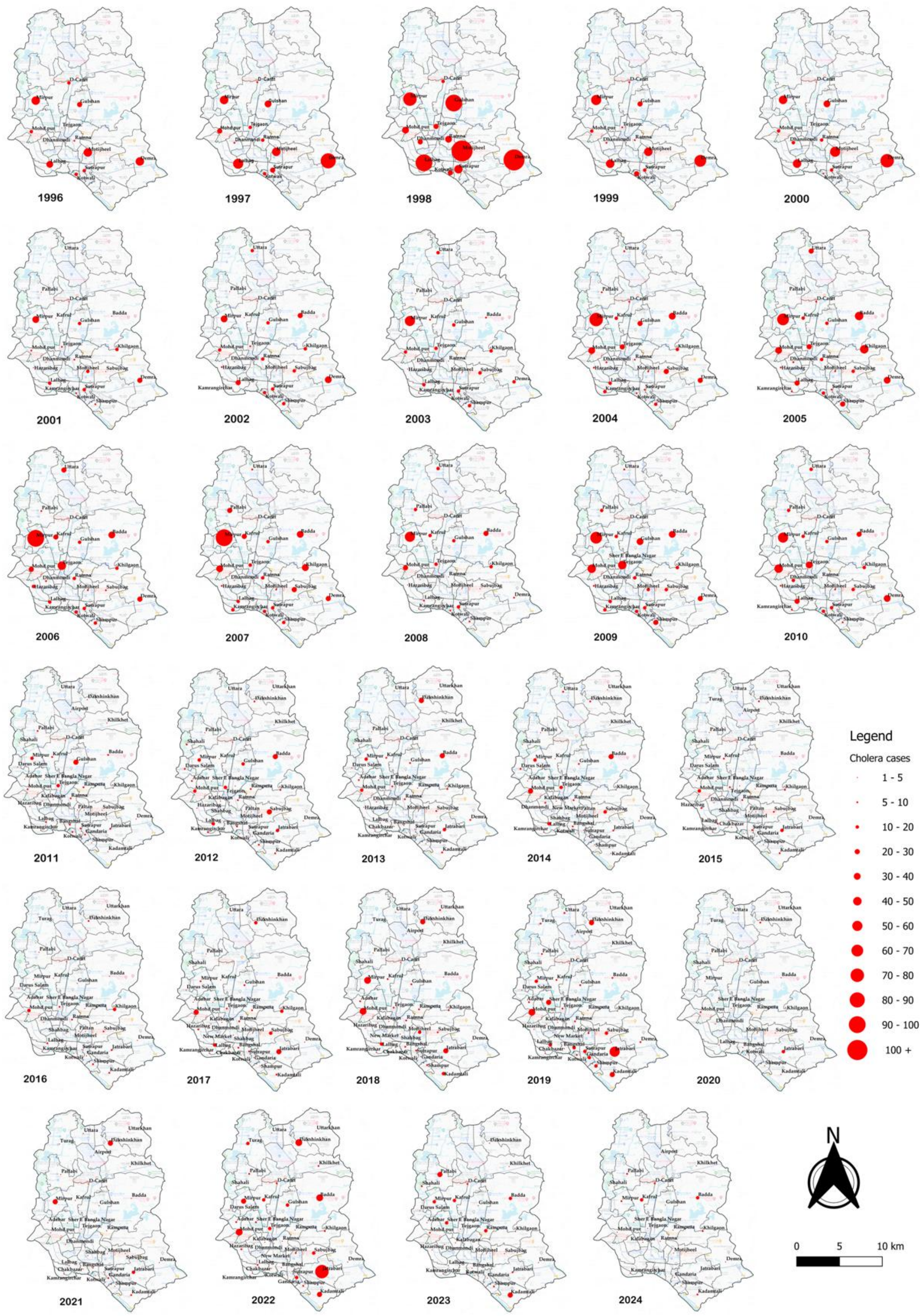

Figure S1: Yearly cholera case distribution in the different parts of the Dhaka city. The figure shows location specific cholera cases in different locations of Dhaka city based on 2% hospital-based surveillance data. Maps were created based on the administrative area of Dhaka city. Maps were created by QGIS Desktop 3.34.3 software based on the administrative area of Dhaka city with EPSG:4326-WGS84-Geographic coordinate reference system (CRS) in the background. The city map (adm-4 level) was collected from the Humanitarian Data Exchange database

(https://data.humdata.org/). The red circle indicates the total cholera cases per year in the respective area, integrated from the icddr,b diarrheal diseases surveillance system. We applied a set of rules to classify the circle size according to the number of cholera cases was shown in the lower right corner of the figure, with the north arrow and scale bar. A total of 41 administrative areas were covered in our study. Several upsurges of cholera were observed in the figure, 1997-1998, 2005-2007, 2009, 2018-19, and 2022. The high number of cholera cases reported in 1998 may be due to the countrywide floods. The period from 1999 to 2003 appeared to be a relatively calm phase, then cholera cases increased in 2004-2005, with a higher number reported in 2006-2007. A drastic reduction in cholera cases occurred after 2010 and continued to 2017. Cholera cases increased slightly in 2018-2019, and after a 3-year gap, a notable cholera outbreak occurred in 2022. Mirpur, Mohammadpur, Jatrabari, Dakshinkhan, and Badda remained as cholera hotspots in most of the upsurges.

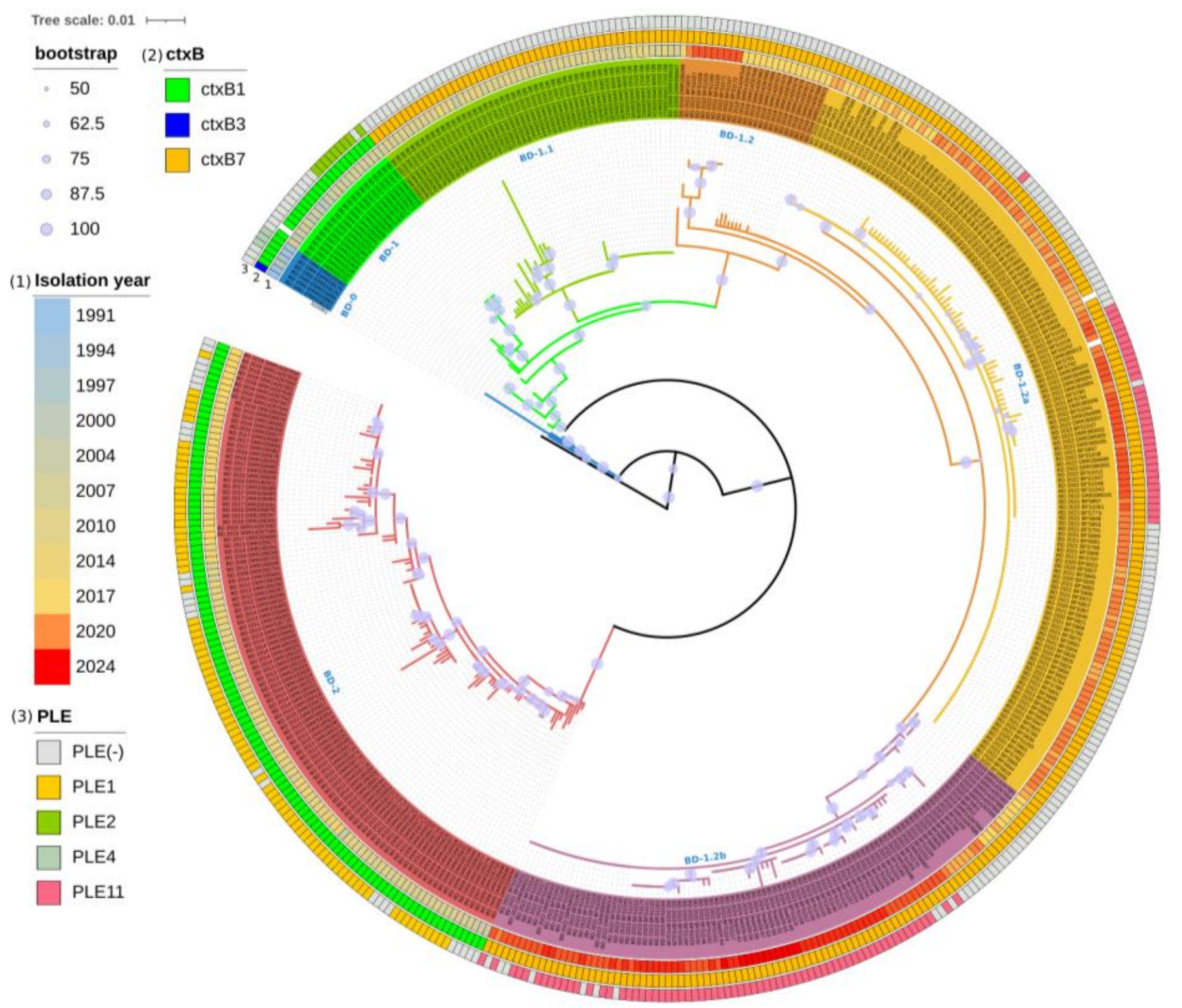

Figure S2: Maximum likelihood based phylogenetic tree constructed for the isolated strains from 1991-2024. Figure shows the maximum likelihood phylogenetic tree visualized with year of isolation, *ctxB* genotype, and phage inducible chromosomal like elements (PLE). Lineages were defined in the similar way as in the previous publication (Monir et al., 2023).

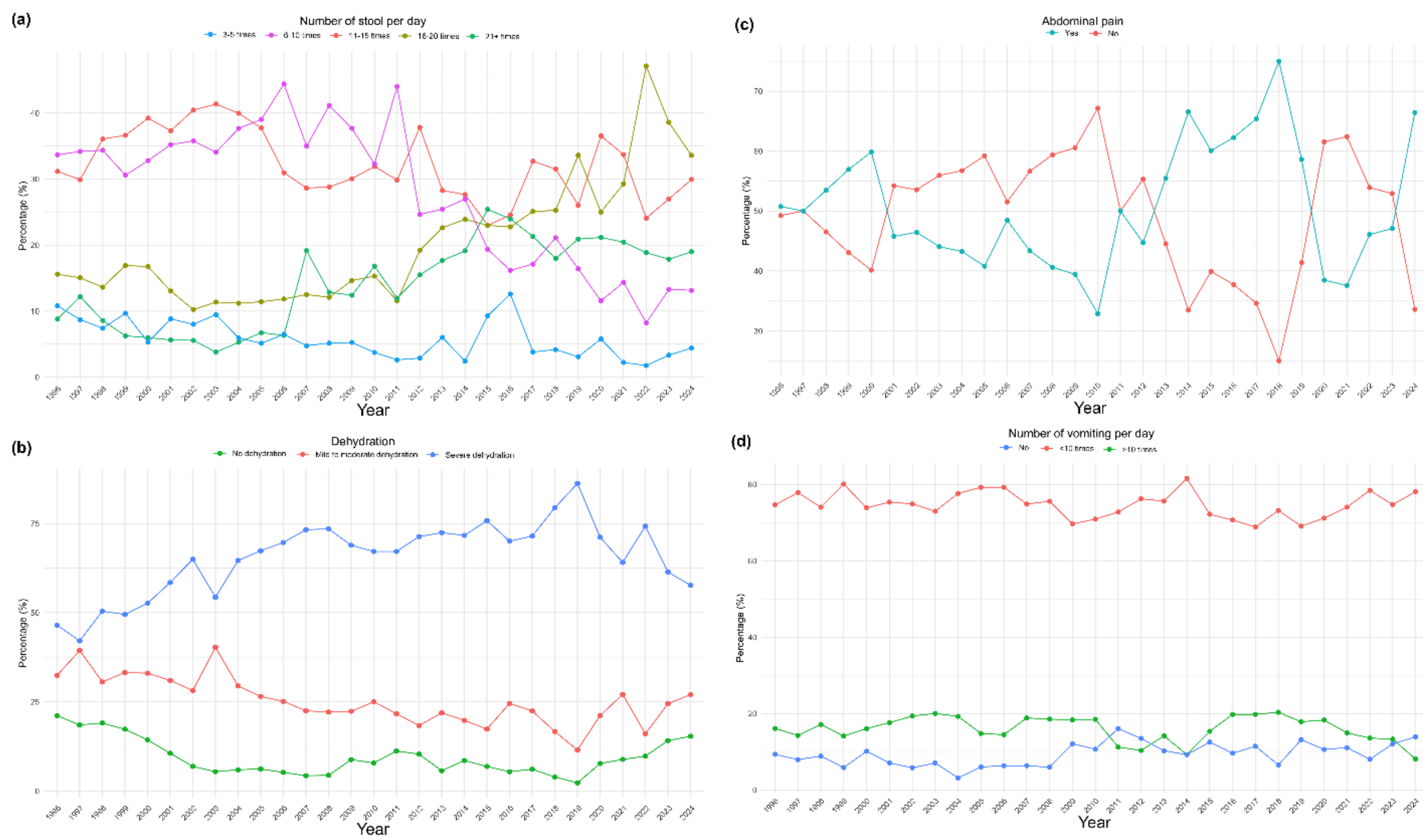

Figure S3: Maximum likelihood based phylogenetic tree constructed for the isolated strains from 1991-2024. (a) number of stools per day increased among cholera patients over time. Patients experiencing 16-20 episodes of diarrhea became more common after 2011, although a slight decrease has been observed in recent years, a large proportion of cases. After 2006, the patients with 21 or more stools per day increased gradually, suggesting a rise in disease severity over time. Patients with 6-10 episodes of diarrhea decreased after 2011. (b) severe dehydrated cholera patients increased gradually over the period, although a decreasing trend was observed in recent years. Mild to moderate dehydrated cholera patients decreased steadily. After 2019, cholera patients with no dehydration have been increasing, but at a very low percentage of patients experienced cholera with no dehydration. (c) Abdominal pain among cholera patients showed temporal variations: an increase was noted from 1996 to 2000, followed by a decline during 2001-2010. Cases rose gradually again between 2011 and 2018, dipped from 2019 to 2021, and have increased in recent years. (d) The major proportion of cholera patients vomited up to 10 times, and a smaller proportion experienced more than 10 episodes of vomiting.

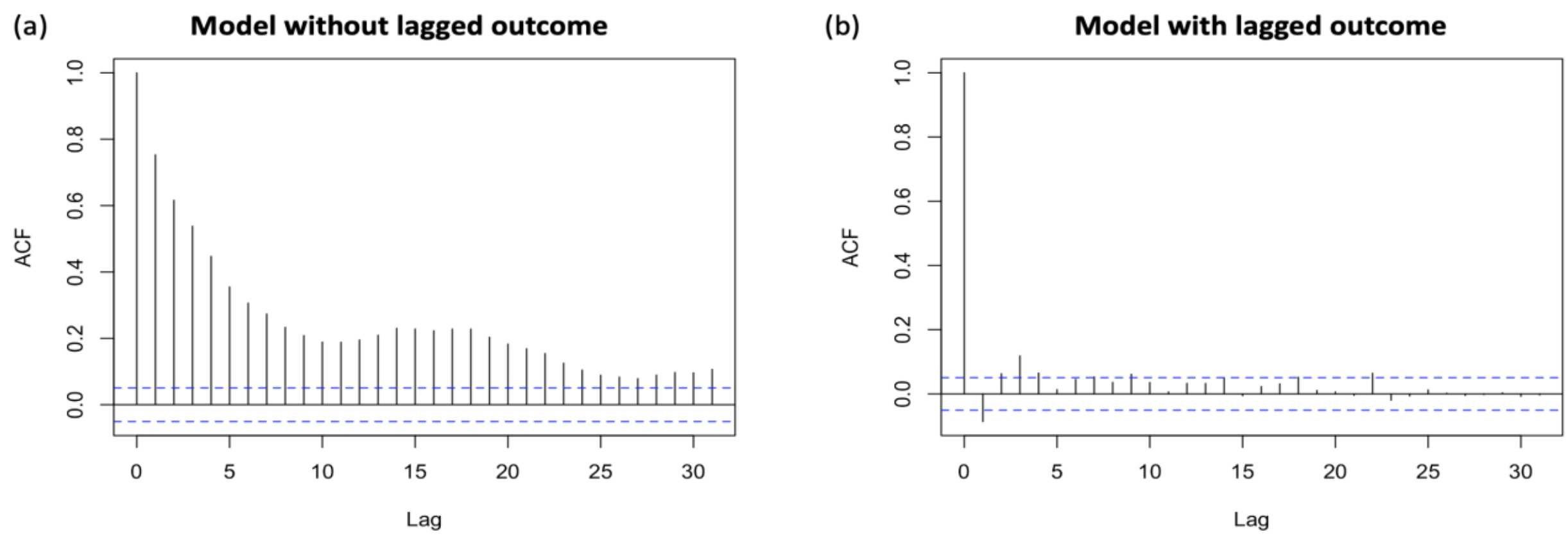

Figure S4. **Autocorrelation functions (ACF) of residuals from generalized additive models with and without a lagged outcome term**.

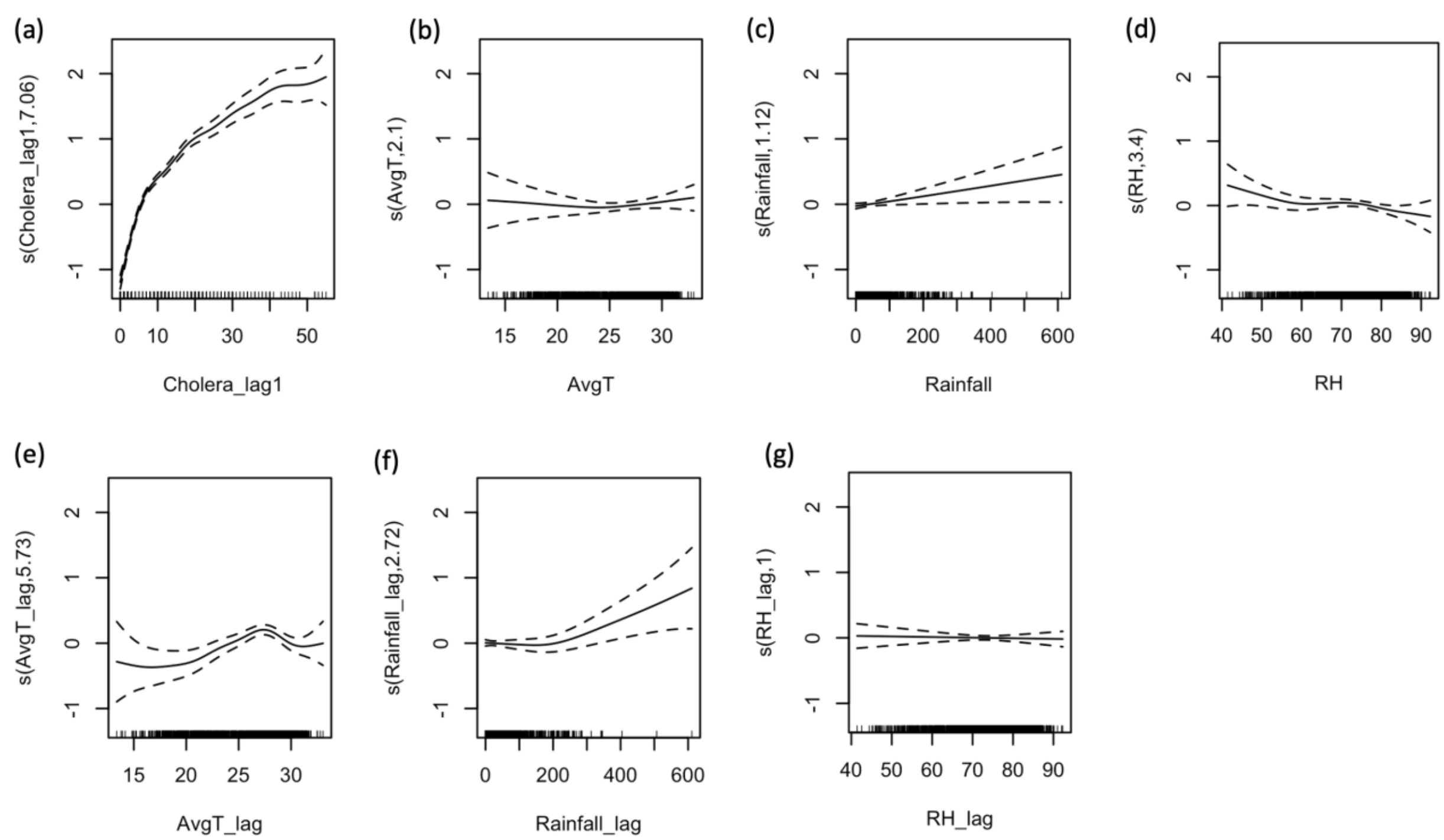

Figure S5. Respecified generalized additive model with climate variables and weekly cholera cases. Here, figures (a-g) show the effects plots based on GAM model for weekly cholera including short term temporal dependence in the outcome, y-axis = estimated effect of smooth function of predictor on outcome (log of cholera cases) and x-axis = predictor variables.
