# Supplementary material for: Genomic evolution and climate related drivers of cholera surges in Dhaka Bangladesh between 1996 and 2024
Source: Commun Med (Lond). 2026 May 22;6:440. doi: 10.1038/s43856-026-01620-8 (PMC13469620; doi:10.1038/s43856-026-01620-8)
Supplement: Supplementary file 2 [file 43856_2026_1620_MOESM2_ESM.docx]

**Description of Additional Supplementary Files**

Supplementary Data 1. List of V. cholerae strains used in this study.

Supplementary Data 2. VSP-2 types identified from the sequence data sets.

Supplementary Data 3. Estimated effect of climatic variables with cholera cases
using GAM model. Here, AvgT refers weekly average temperature; Rainfall refers
weekly total rainfall; RH refers weekly average relative humidity; AvgT_lag refers
average temperature in the preceding week; Rainfall lag refers total rainfall in the preceding week; RH_lag refers average relative humidity in the
preceding week; and s() refers smoothing function capturing non-linearity.

Supplementary Data 4. Modelling yearly cholera cases with climate and genomic
evolutionary variables using negative binomial regression.

Supplementary Data 5. Lineage associated genetic variants.

Supplementary Data 6. Confirmed cholera cases in the years 1996-2010 and 2011-
2024.

Supplementary Data 7. Confirmed cholera cases in the years 1996-2010 and 2011-
2024.

Supplementary Data 8. Summary statistics of Summary statistics of cholera positive patients’ factors.
